# Engineering serendipity: When does knowledge sharing lead to knowledge production?

**DOI:** 10.1002/smj.3256

**Published:** 2020-11-24

**Authors:** Jacqueline N. Lane, Ina Ganguli, Patrick Gaule, Eva Guinan, Karim R. Lakhani

**Affiliations:** ^1^ Laboratory for Innovation Science at Harvard Harvard Business School Boston Massachusetts USA; ^2^ Department of Economics University of Massachusetts Amherst Amherst Massachusetts USA; ^3^ Department of Economics University of Bath Bath UK; ^4^ Dana‐Farber Cancer Institute Boston Massachusetts USA

**Keywords:** innovation, knowledge production, knowledge sharing, knowledge similarity, natural field experiment

## Abstract

**Research Summary:**

We investigate how knowledge similarity between two individuals is systematically related to the likelihood that a serendipitous encounter results in knowledge production. We conduct a field experiment at a medical research symposium, where we exogenously varied opportunities for face‐to‐face encounters among 15,817 scientist‐pairs. Our data include direct observations of interaction patterns collected using sociometric badges, and detailed, longitudinal data of the scientists' postsymposium publication records over 6 years. We find that interacting scientists acquire more knowledge and coauthor 1.2 more papers when they share some overlapping interests, but cite each other's work between three and seven times less when they are from the same field. Our findings reveal both collaborative and competitive effects of knowledge similarity on knowledge production outcomes.

**Managerial Summary:**

Managers often try to stimulate innovation by encouraging serendipitous interactions between employees, for example by using office space redesigns, conferences and similar events. Are such interventions effective? This article proposes that an effective encounter depends on the degree of common knowledge shared by the individuals. We find that scientists who attend the same conference are more likely to learn from each other and collaborate effectively when they have some common interests, but may view each other competitively when they work in the same field. Hence, when designing opportunities for face‐to‐face interactions, managers should consider knowledge similarity as a criteria for fostering more productive exchanges.

## Abstract 2

https://www.youtube.com/watch?v=c7gCUtYb0dQ

## 1 INTRODUCTION

In 2013, cell biologist William Earnshaw of the University of Edinburgh happened to attend the same academic conference as systems biologist Job Dekker of the University of Massachusetts Medical School and computational biologist Leonid Mirny from the Massachusetts Institute of Technology. By chance, Earnshaw attended a presentation by Dekker and Mirny about their joint work on mitotic chromosomes, during which he became convinced that his lab could provide bench methods to improve Dekker and Mirny's computational models. Earnshaw approached Dekker and Mirny after the talk; their conversation evolved into a three‐lab collaboration and a 2018 publication in *Science* (Pain, 2018).

This anecdotal example suggests that serendipitous encounters can play a role in innovation, perhaps by exposing individuals to people that they would not otherwise have a chance to meet, and to unfamiliar sources of information that can be combined with their own knowledge stock and lead to new discoveries (Fleming, Mingo, & Chen, 2007; Uzzi, Mukherjee, Stringer, & Jones, 2013). However, the likelihood that serendipitous encounters lead to successful knowledge production remains poorly understood often because the process is uncertain, complex and relatively understudied. In particular, even if an encounter eventually produces new knowledge, this process may occur slowly and only emerge after a number of years (Catalini, 2018; Fleming, 2001). A significant degree of knowledge within organizations is tacit and is not easily transferred (Hansen, 1999; Nonaka, 1994). Knowledge creation via new collaborations can be costly (Boudreau et al., 2017; Dahlander & McFarland, 2013), requiring coordinated effort, alignment of incentives, establishment of trust, generation of creative synergy, and “matching” criteria, such as personality and scheduling compatibility (Azoulay, Ding, & Stuart, 2007; Catalini, 2018). Even if knowledge transfer or creation is successful, knowledge tends to diffuse slowly through interpersonal networks (Fleming et al., 2007; Singh, 2005). The question thus arises of when serendipitous encounters are more likely to facilitate useful idea exchange and knowledge production. Previous literature tends to focus on the factors that may increase the amount or intensity of short‐term communication and interaction between individuals, rather than the implications of serendipitous encounters on knowledge production (Allen, 1977; Kleinbaum, Stuart, & Tushman, 2013). Given that geographic proximity increases the likelihood of serendipitous encounters (Catalini, 2018), recent research has begun to examine the effects of organizational redesign on knowledge production (Catalini, 2018; Fang, Lee, & Schilling, 2010; Lee, 2019). Although it is possible that organizational redesign and greater geographic proximity between individuals can increase knowledge production, most studies do not directly address how knowledge sharing between two individuals affects the path‐dependent nature of the knowledge production process.

Since prior work tends to focus on a single knowledge production outcome (Phelps, Heidl, & Wadhwa, 2012), often on the factors that impede knowledge transfer (Carlile, 2004; Hansen, 1999; Szulanski, 1996; Uzzi, 1997), it is unclear how knowledge sharing may also affect knowledge creation and diffusion. Hence, we know relatively little about whether serendipitous encounters actually lead to meaningful idea exchange that can be applied to one's own tasks, or whether these interactions can spark new collaborations and broader diffusion of ideas over time. The goal of our work is to address this gap in literature by examining to what extent a systematic relationship exists between serendipitous encounters and knowledge production outcomes.

We posit that an effective encounter hinges not just on the serendipity of the interaction but also on the existence of common knowledge that individuals use to make sense of each other's specialized knowledge and connect it to what they already know. Common knowledge enhances the ability for new knowledge to be assimilated into the concepts, objects and patterns that are already present in people's cognitive structures (Bower & Hilgard, 1981; Cohen & Levinthal, 1990). When new information is related to prior knowledge constructs, it is more readily absorbed, integrated, and applied in new settings (Carlile, 2004; Kogut & Zander, 1992) without sufficient prior knowledge, individuals may have difficulty integrating new knowledge (Rosenkopf & Almeida, 2003). In this article, we argue that there are two essential characteristics to people's common knowledge: *field similarity* in their educational backgrounds and training, and *intellectual similarity* in their interests, passions and pursuits. Serendipitous encounters are more likely to be effective when people share some knowledge similarity in both their field and intellectual interests.

To systematically investigate the relationships between serendipitous encounters and knowledge production requires a rich longitudinal dataset that directly observes exogenous face‐to‐face knowledge sharing between two individuals and subsequent knowledge production outcomes. To draw causal inferences, we designed and executed a field experiment at a research symposium on advanced imaging in early 2012 at a leading academic medical center. We chose this setting due to the centrality of knowledge production to their organizational and individual performance goals and the prevalence of knowledge sharing norms (Dahlander & McFarland, 2013). We created exogenous variation in opportunities for information‐rich, face‐to‐face encounters between cross‐disciplinary scientists interested in applying for an internal grant program promoting the development of advanced imaging solutions to address an unmet clinical need. Using electronic sociometric badges (Kim, McFee, Olguin, Waber, & Pentland, 2012), we collected a unique dataset of fine‐grained, live interactions between pairs of scientists who were randomly assigned to be in the same symposium room for knowledge sharing. We then combined this with a rich longitudinal dataset on long‐run knowledge production outcomes from the scientists' written publications over a six‐year period, where we linked scientists' pairwise keywords, collaborations and forward citations to examine the extent that the encounters led to knowledge transfer, creation and diffusion, respectively. This design enabled us to causally and systematically identify the relationships between knowledge sharing and knowledge production to explain why some serendipitous encounters resulted in knowledge production while others did not.

We find both cooperative and competitive effects of serendipitous knowledge sharing encounters on knowledge production. On the one hand, knowledge sharing led to greater knowledge transfer and knowledge creation when people had some overlapping intellectual and field interests, respectively. Although knowledge sharing had limited impact on knowledge transfer among pairs with either few or many overlapping interests, we observe a twofold increase in knowledge transfer (of Medical Subject Headings or MeSH keywords) for moderately similar pairs, suggesting an inverted U‐shaped relationship between knowledge overlap and knowledge transfer. Similarly, we find that knowledge sharing between pairs with some common interests led to 1.2 more coauthored peer‐reviewed publications, compared to a 0.98 decrease in copublications among pairs in the same field, which suggests that the knowledge sharing intervention reduced the search costs associated with forming synergistic collaborations. By comparing the knowledge drivers of near‐term collaborations and long‐term collaborations, we find that common knowledge is more critical for the former. On the other hand, knowledge sharing reduces knowledge diffusion between people from the same field, with interacting pairs citing each other between 2.8 and 6.7 times less than noninteracting pairs from more distant fields.

We aim to make several contributions to the literature. First, we contribute to the literature on search costs and information frictions associated with the knowledge production process (Agrawal & Goldfarb, 2008; Boudreau et al., 2017; Catalini, 2018; Hansen, 1999; Hansen & Haas, 2001), by focusing on the extent that “engineered serendipity” can mitigate some of these costs. We emphasize the role of knowledge similarity in shaping different components of knowledge production, and over different time horizons. Most prior research has predominantly focused on one component or a single time frame (Adams, Black, Clemmons, & Stephan, 2005; Boudreau et al., 2017; Dahlander & McFarland, 2013; Szulanski, 1996). This work is unique because it extends the findings from Boudreau et al. (2017) on search costs and short‐term collaborations using the same field experiment, to show how engineered serendipity may alter the role of knowledge similarity in knowledge production over time. Our findings suggest that the magnitude of these frictions is likely to be more difficult to overcome in the short‐run compared to the long‐run.

Second, we make contributions to the literature on temporary colocation, and the value of conferences, symposia and similar events on the direction of inventive activity on the knowledge frontier and the emergent patterns of collaborative activity (Biancani, McFarland, & Dahlander, 2014; Boudreau et al., 2017; Campos, Leon, & McQuillin, 2018; Catalini, Fons‐Rosen, & Gaulé, 2020; Chai & Freeman, 2019). Prior research has been limited in its ability to observe actual acts of knowledge sharing between pairs, and its implications on knowledge production. Our study indicates that even brief, information‐rich encounters at conferences can benefit knowledge production, with the potential to alter people's research directions and collaboration networks.

Third, we make methodological contributions by highlighting the benefits of long‐term studies that amalgamate multiple forms and uses of data. Prospective experiments can support multiple lines of investigation involving both near‐term and long‐term outcomes that may not be possible in retrospective, archival studies and suggests the use of multiple sources of data for unpacking the dynamics of knowledge production.

## 2 THEORY AND HYPOTHESES

### 2.1 Knowledge production and knowledge sharing

According to the knowledge‐based view of the firm, knowledge production is a multicomponent process that involves the transfer, creation and diffusion of knowledge (Grant, 1996; Kogut & Zander, 1992; Nonaka, 1994). Knowledge transfer involves the movement of facts, relationships, and insights from one setting to another, and becomes evident when the experience acquired by an individual, group or organization in one setting is applied in another setting (Argote, 2012; Hansen, 1999; Szulanski, 1996). Knowledge creation refers to the generation of facts, relationships and insights to solve new problems (Nonaka, 1994). The emphasis on *new* knowledge is what distinguishes the process of knowledge transfer from creation. Because new knowledge is not held by anyone prior to its creation, it cannot be transferred and applied directly (McFadyen & Cannella Jr, 2004). Conversely, knowledge diffusion refers to the process through which transferred or newly created knowledge is disseminated and used by other individuals, groups and organizations (Fleming et al., 2007; Singh, 2005). Knowledge diffusion is a critical component of the knowledge production process because it reduces duplication of effort and promotes efficiency by demarcating what is known from what is yet to be explored on the knowledge frontier (Boudreau & Lakhani, 2015).

Critical to the efficiency of knowledge production is the process of knowledge sharing between individuals possessing different types of knowledge (Grant, 1996; Kogut & Zander, 1992; Nonaka, 1994), and their ability to learn from these interactions (Levinthal & March, 1993; Simon, 1991). Common knowledge between partners is important because it enables individuals to absorb the aspects of their knowledge sets that they do not hold in common, or that are unique to each individual (Carlile, 2004; Cohen & Levinthal, 1990; Maurer & Ebers, 2006). Building on prior work, we propose that there are at least two separate pathways through which people develop their knowledge bases: their field and intellectual specialties. Because of the path‐dependent nature of knowledge (Hargadon & Sutton, 1997), the overlap in two individuals' field and intellectual specialties may have different implications on knowledge production. Table 1 provides a summary of our knowledge constructs.

**TABLE 1:** Summary of knowledge constructs

Knowledge construct	Definition	Citations
Knowledge production	A multicomponent process that involves knowledge transfer, creation, and diffusion.	Grant, 1996; Kogut & Zander, 1992; Nonaka, 1994; Spender, 1996
Knowledge transfer	The movement of facts, relationships, and insights from one setting to another	Argote, 2012; Hansen, 1999; Szulanski, 1996
Knowledge creation	The generation of facts, relationships, and insights to solve problems that are new to the knowledge frontier	Arrow 1962; Boudreau & Lakhani, 2015; Nonaka, 1994
Knowledge diffusion	The dissemination of facts, relationships, and insights that are subsequently used by others	Fleming & Singh, 2010; Singh, 2005
Knowledge similarity	The degree of common knowledge between two individuals' field of study and intellectual interests	Cohen & Levinthal, 1990; Carlile, 2004; Dahlander & McFarland, 2013; Leahey, Beckman, & Stanko, 2017
Field similarity	The dimension of knowledge similarity that emerges from the overlap between two individual's educational background, skills, and training	Bechky, 2011; Carlile, 2004; Bourdieu, 1984; Haas & Park, 2010
Intellectual similarity	The dimension of knowledge similarity that emerges from the overlap between two individual's personal interests and passions	Caza, Moss, & Vough, 2018; Dahlander & McFarland, 2013

### 2.2 Two knowledge bases of similarity: Field specialty and intellectual specialty

Field specialties are developed through an individual's educational background, skills and training (Bechky, 2011; Bourdieu, 1984; Haas & Park, 2010). In biomedical research, which is our focal context of study, there are multiple medical field specialties, such as neurology, radiology or oncology that focus on investigations of the biological process and the causes of specific diseases. Organizations often structure their departmental and divisional memberships around field specialties (Biancani et al., 2014). In universities and academia, this means that field specialties are often the primary channels through which resources, such as salaries, funding, tenure, offices and laboratory space, are apportioned to faculty (Biancani, Dahlander, McFarland, & Smith, 2018). For these reasons, field specialties provide professional reference groups (Haas & Park, 2010) for members to identify appropriate behavior, rigor of scholarship, career goals and aspirations (Abbott, 2001). In contrast, intellectual specialties are closely aligned to personal interests and passions that evolve over time. Individuals can have memberships in multiple intellectual specialties depending on their current pursuits and priorities (Caza et al., 2018). For example, in biomedical research, intellectual specialties include research topics such as aging, addictions, Alzheimer's disease, brain injury, sleep, and stem cells; each of these intellectual specialties is associated with memberships in professional reference groups (Haas & Park, 2010) for people motivated by common problems, methods, trends, and processes. These intellectual interests can be specific to a field's research topics, such as Alzheimer's disease, which is typically studied by neurologists, or span different fields, such as stem cell research.

In short, field and intellectual specialties shape people's identities, language, tastes and affiliations through professional memberships (Haas & Park, 2010). Individuals will likely adopt multiple professional identities depending on their field and intellectual memberships: this means that people from different field specialties can share overlapping intellectual interests, while individuals from the same field specialty can have divergent intellectual interests, because field and intellectual interests are two distinct dimensions of individuals' knowledge constructs.

In distinguishing how field and intellectual overlap shape knowledge production, knowledge similarity describes the distance between the field specialties and intellectual specialties of two knowledge sharing partners. Individuals are more likely to recognize and absorb new ideas when they already have some existing expertise and find it more difficult when the ideas are outside their realm of expertise (Carlile, 2004; Cohen & Levinthal, 1990). Put differently, even though distant knowledge tends to be novel and valuable (Jeppesen & Lakhani, 2010; Leahey et al., 2017), people are more likely to experience challenges communicating with one another, and may not recognize the value of external information (Cohen & Levinthal, 1990; Grant, 1996). For example, two neurologists are less likely to experience challenges communicating with each other than a neurologist and oncologist pair because they share more similar educational backgrounds, training and clinical expertise. However, there may be greater opportunities for knowledge production between the neurologist and oncologist pair because their field specialties are more distant. Similarly, although two sleep researchers may communicate and comprehend each other with relative ease because they read and conduct similar research (Dahlander & McFarland, 2013), there is greater potential for knowledge production between an aging and a sleep researcher, who may offer greater utility and value to one another to solve problems related to both sleep and aging processes—provided that they have sufficient common knowledge (Bechky, 2011; Grant, 1996). This suggests that there is an inherent trade‐off between the greater ease of conversing and absorbing ideas locally from individuals with many overlapping interests and the greater potential opportunities for novel ideas and knowledge transfer, creation, and diffusion from sharing and assessing the knowledge of those with more distant interests.

### 2.3 Knowledge transfer and knowledge similarity

Knowledge transfer is a two‐sided process because it depends on the efforts of a source to share knowledge with a recipient and the recipient's efforts and capacity to acquire, absorb, and learn it (Argote, 2012). Because of greater common knowledge, local knowledge found within groups of similar individuals tends to be more easily transferred than distant knowledge spanning group boundaries (Carlile, 2004; Kogut & Zander, 1992; Rosenkopf & Almeida, 2003). That said, more distant knowledge may present nonredundant ideas that benefit learning and acquiring new concepts. Consequently, during serendipitous idea exchanges, we would expect that partners from dissimilar field and intellectual specialties offer each other more novel ideas and opportunities to learn (Leahey et al., 2017; Lee, 2019). Divergent interests create a wider pool of knowledge to draw upon, which allows for multiple perspectives and problem‐solving approaches that increase the likelihood of new discoveries (Boudreau, Guinan, Lakhani, & Riedl, 2016)—provided that the partners share sufficient common knowledge to make sense of each other's knowledge dissimilarities. Based on these reasons, we expect that pairs with less field or intellectual overlap have a greater potential pool of ideas for knowledge transfer, up until a threshold of dissimilarity, after which they will lack sufficient common interests to benefit from knowledge transfer.

Hypothesis (H1a) *Field similarity has an inverted U‐shaped effect on the relationship between knowledge sharing and knowledge transfer*.

Hypothesis (H1b) *Intellectual similarity has an inverted U‐shaped effect on the relationship between knowledge sharing and knowledge transfer*.

### 2.4 Knowledge creation and knowledge similarity

There is a growing view among innovation scholars that specialization has created a “knowledge burden” hypothesis that makes collaborative work combining the increasingly narrow niches of specialization imperative to moving the knowledge frontier forward (Jones, 2009; Uzzi et al., 2013). Scientific discovery is a process that combines individually focused tasks—such as reading, experimentation, and writing—with social interactions through joint sense‐making with others that can spark new discoveries (Boudreau et al., 2016; Latour & Woolgar, 2013).

That said, collaborations tend to be constrained by social processes, such as preferences for others with shared attributes, as well as search costs that stifle attempts to identify suitable collaborators. First, principles of homophily suggest that people are attracted to others who hold similar values because their interactions are more rewarding and less uncertain (McPherson & Smith‐Lovin, 1987), as a result of the benefits of security and mutual attraction (Dahlander & McFarland, 2013). Moreover, joint knowledge creation is a long‐term investment in a multiplex relationship (Uzzi, 1997), where partners engage in joint problem‐solving and spend time together discussing, reflecting and interacting to achieve mutual benefit (McFadyen & Cannella Jr, 2004). Due to these reasons, collaborations are more likely to form among individuals who share overlapping field and intellectual interests (Biancani et al., 2014; Dahlander & McFarland, 2013).

Second, there is a growing view that search costs and resulting information frictions tend to constrain people's collaboration patterns (Boudreau et al., 2017; Catalini, 2018). Engineered, serendipitous interactions can mitigate some of these frictions by providing people the opportunity to exchange information and develop intellectual links with others they would not otherwise interact with. Such interactions may enable diverse partners to discover the potential complementarities they offer each other, leading to new collaborations. However, to the extent that serendipitous knowledge sharing leads to joint knowledge creation will also likely require that potential partners share some field or intellectual overlap; otherwise their interests may be too disparate to establish synergies in incentives (e.g., publication or tenure requirements) or research interests. Accordingly, we expect that increasing field and intellectual similarity should benefit knowledge creation up to a threshold, over which there are decreasing marginal returns on greater levels of knowledge similarity.

Hypothesis (H2a) *Field similarity has an inverted U‐shaped effect on the relationship between knowledge sharing and knowledge creation*.

Hypothesis (H2b) *Intellectual similarity has an inverted U‐shaped effect on the relationship between knowledge sharing and knowledge creation*.

Another pathway that may lead to joint knowledge creation relies on prior collaborations and tie persistence (Hasan & Koning, 2019; Ingram & Morris, 2007; Zhang & Guler, 2020). We examine a specific type of tie persistence, namely the likelihood that an elemental collaboration persists into a complete knowledge product. In science, grant coapplications and copublications represent two essential but opposing ends of knowledge creation, from idea‐generation to final output (McFadyen & Cannella Jr, 2004). Both are also essential to scientists' research productivity and often used as evaluative criteria for important decisions, such as promotion, tenure, awards and recognition (Dahlander & McFarland, 2013; Stephan, 1996). The knowledge similarity requirements that improve the likelihood that elemental collaborations persist into copublications may differ from the processes that shape long‐term collaborations on peer‐reviewed research publications. In addition to being at the open end of research inquiry, grant applications tend to have finite submission deadlines and these can impose resource, attention, coordination and other constraints on potential partners (Dahlander, O'Mahony, & Gann, 2016). Although serendipitous knowledge exchanges can reduce information search costs and expose people to promising new contacts, multiplex relationships require repeated interactions (Ingram & Morris, 2007). In the short‐term, potential partners may turn to other individuals with whom they share significant knowledge overlap, due to fewer frictions and relational uncertainties (e.g., priorities, personalities, scheduling constraints) associated with similar partners. Due to these reasons, common knowledge may be even more critical to shaping elemental collaborations. We thereby expect that elemental collaborations are more likely to persist into a final product of knowledge creation when knowledge sharing partners have significant knowledge overlap in terms of their field and intellectual interests.

Hypothesis (H2c) *An elemental collaboration is more likely to result in knowledge creation as the field similarity between two individuals increases*.

Hypothesis (H2d) *An elemental collaboration is more likely to result in knowledge creation as the intellectual similarity between two individuals increases*.

### 2.5 Knowledge diffusion and knowledge similarity

Among research scientists, forward citations to others' publications are a primary means for diffusing knowledge. Beyond knowledge diffusion, forward citations constitute a critical means of social recognition for acknowledging the contributions of predecessors (Merton, 1973) and tracing the path of scientific discovery and diffusion (Stephan, 1996). Some scholars argue that the number of citations a publication has received is perhaps the most common way to measure the importance of an individual's contribution to science (Stephan, 1996). Consistent with this view, citations are a critical currency of scientific credit (Latour & Woolgar, 2013), both driving research impact and constituting the basis of reward systems in science, including promotion, status, funding, peer esteem, honors, and awards (Boudreau & Lakhani, 2015).

There are generally two views of how knowledge is diffused in science: openness and secrecy. According to the Mertonian norms of communalism or “openness,” publication enables scientists to establish priority of discovery and allows them to be the first to communicate an advance in knowledge and allow others to freely use it (Merton, 1973). Thus, publication promotes the open diffusion of scientific knowledge, as long as scientists' own internal agents (i.e., other scientists) appropriately recognize and diffuse their work (Boudreau & Lakhani, 2015). On the other hand, social recognition is a discretionary act among scientists, and strong evidence points to the existence of counter‐norms promoting secrecy, competition, and information withholding (Haas & Park, 2010; Haeussler, Jiang, Thursby, & Thursby, 2014). Scientists often compete for similar resources, funding, and recognition. Given limited resources, scientists need to be assured that they will be appropriately remunerated for openly diffusing others' knowledge (Hagstrom, 1974; Murray & O'Mahony, 2007; Reschke, Azoulay, & Stuart, 2018).

On balance, the evidence suggests that scientists do not unequivocally diffuse each other's publications. Among scientists with greater knowledge overlap, serendipitous idea sharing may heighten the competition effect, particularly as information exchange can introduce people to novel work they cannot discover on their own via publications or other publicly available sources. These new projects may be similar to their own undertakings, risking duplication of efforts. Because scientists compete for priority (Merton, 1973), recognition (Hagstrom, 1974; Reskin, 1977), and funding (Stephan, 1996), partners with highly overlapping interests may discover a need to differentiate themselves from one another. Social recognition of highly similar peers would not only highlight redundancies but may also detract attention and resources away from one's own work (Campanario & Acedo, 2007). We expect that partners sharing high field or intellectual similarity may refrain from citing each other's research to outperform their peers.

Hypothesis (H3a) *Knowledge sharing is less likely to lead to knowledge diffusion as the field similarity between individuals increases*.

Hypothesis (H3b) *Knowledge sharing is less likely to lead to knowledge diffusion as the intellectual similarity between individuals increases*.

## 3 EXPERIMENTAL METHODS

In an ideal setting, all of the prior interactions and efforts that go into knowledge production would be fully observable to scholars to theorize and validate through empirical observations. The reality, however, is that the vast majority of prior scholarly work concerned with how knowledge is produced has primarily focused on *observed* outcomes—e.g., papers, funding, patents, citations, team structure, and so forth—to draw inferences about the mechanisms underlying the knowledge production process (Dahlander & McFarland, 2013; Fleming et al., 2007; Staw & Epstein, 2000). For example, research on scientists will include the papers they publish, the collaborators they have worked with, and the knowledge that they have developed and have diffused through citations. However, a concern with relying on published trace data is that it masks all of the work and activities that occurred prior to knowledge production, such as the entire risk set of alters an individual may have interacted with prior to settling on a particular team. The empirical shortcomings of not being able to directly observe knowledge sharing and the drivers of knowledge production may insert biases in our inferences, such as self‐selection and survivor bias. Some work aims to address these concerns by extending the risk set to unsuccessful collaborations (e.g., non‐awarded grant applications) to examine quality‐adjusted research output of published (i.e., visible) trace data (Arora & Gambardella, 2005; Ayoubi, Pezzoni, & Visentin, 2019; Ganguli, 2017). That said, unobserved heterogeneity between the two groups remains a persistent challenge.

A feasible alternative is to design a field experiment that enables the capture of data around interactions between scientists and overcomes concerns around endogeneity of affiliation, team formation, and knowledge exposure by randomizing the encounters that the scientists have with each other. The benefit of this approach is that it can provide causal explanations about the factors that impact knowledge production, weighed against the challenges of drawing inferences with smaller sample sizes in an experiment as opposed to relying on all observed data of many more scientists.

### 3.1 Setting and research design

We carried out our study in the context of a medical symposium for research on advanced imaging, which is used to detect diseases and other health conditions early, to allow health care practitioners to direct patients to the health care services that they need. We collaborated with the administrators of a large U.S. medical school to layer the medical symposium onto the University's Clinical and Translational Science Center pilot grant program, which provides seed funding in the form of pilot grants to support nascent research efforts that are awarded competitively to faculty within the University.^1^ The purpose of the grant opportunity was to solicit proposals to improve methods for using advanced imaging technologies to address unmet clinical needs, and offered $50,000 per award for up to 15 pilot grants. A major challenge in the field of advanced imaging is that furthering the knowledge frontier requires expertise in the latest imaging tools and technologies and a deep understanding of the health problems to which they could be applied. These different types of knowledge are typically held by people from distinct disciplinary backgrounds, which makes advanced imaging an ideal setting for our research.

In November 2011, we invited all life sciences faculty and researchers at the university to a medical research symposium for the unique grant funding opportunity. Our field experiment involved faculty and researchers at the University and its affiliated hospitals and institutions, which are independently owned and managed, with each appearing as a separate entity in hospital rankings and lists of National Institutes of Health (NIH) grant recipients. We communicated to applicants that eligibility to submit a grant application was conditional on attending the symposium. In the first stage, investigators interested in applying for the grants submitted a statement of interest in which they briefly described a specific medical problem that advanced imaging techniques could potentially address. We collected basic biographical information (e.g., degree, institution, department appointment) at this stage.

### 3.2 Participants and randomization of knowledge‐sharing partners

**FIGURE 1:**
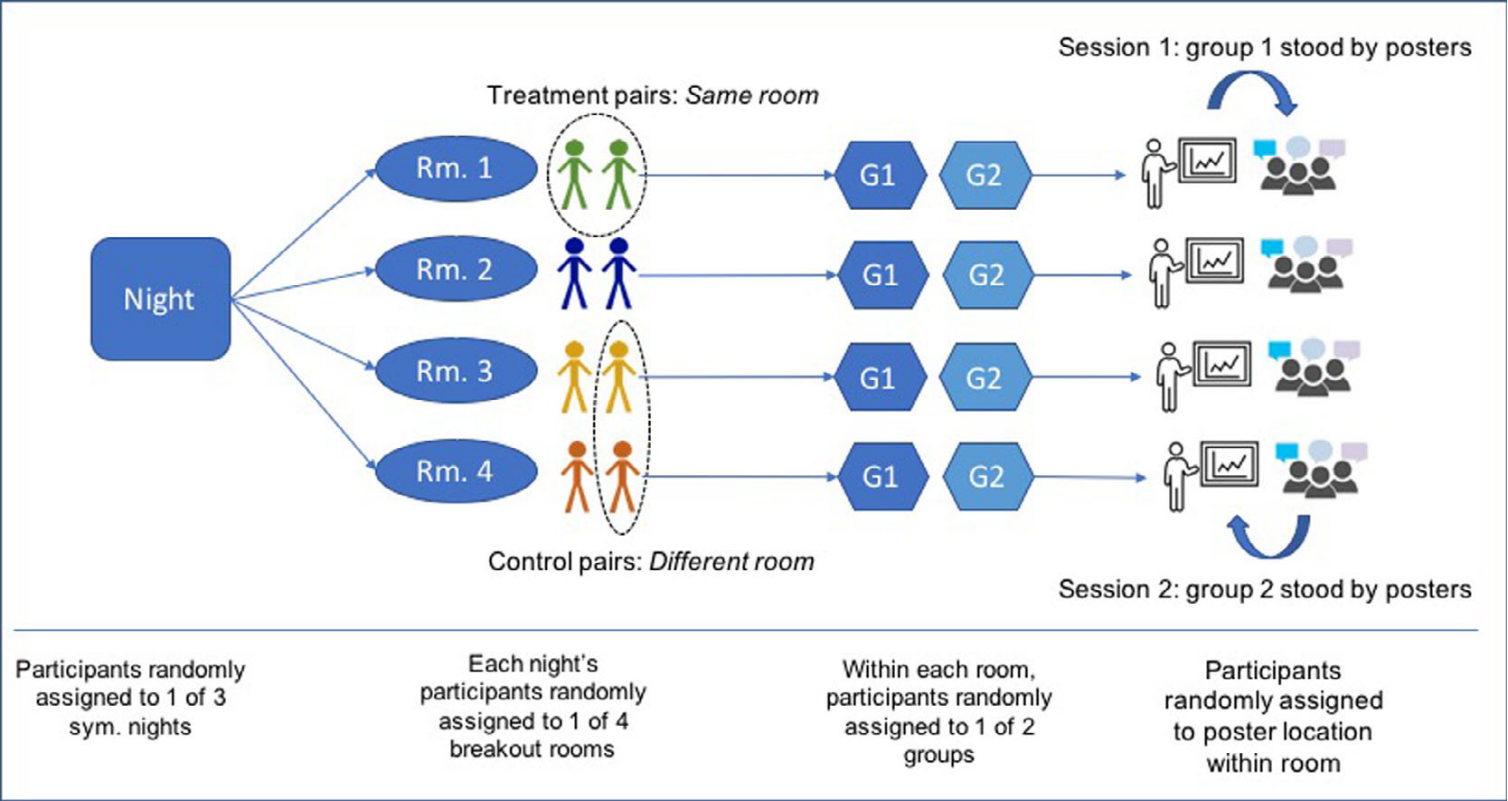
Randomization of participants by night, room, group, and poster location

The symposia were held on January 31, February 1, and February 2, 2012, at one of the university's innovation labs. Figure 1 summarizes the key details of the randomization: 402 unique participants were randomly assigned to one of three nights of the symposium, one of four breakout rooms, one of two groups, as well as a poster location to stand next to around the perimeter of the room.^2^ Participants were provided an electronic device, called a “sociometric badge” to automatically record their face‐to‐face interactions during the symposium (Kim et al., 2012). Each of the three nights featured a 30‐min welcome address, followed by two 45‐min poster sessions in breakout rooms, with a 15‐min social break in between the poster sessions. Within each breakout room, scientists were randomly assigned to a poster location and either the first or second poster group. The scientists presented and exchanged ideas with one another during the poster sessions. The grant administrators prepared the posters to be a standard size and in a standard format, based on each participant's statements of interest. Thus, treatment pairs assigned to the same breakout rooms had more opportunities for knowledge sharing than control pairs.

Shortly after the symposia, all participants received an e‐mail invitation to submit applications for the pilot grants or concept awards by the deadline of March 8, 2012. At this time, they also received PDF booklets with the contact information and posters of all participants so that all researchers had identical information apart from the knowledge acquired in the breakout rooms.

### 3.3 Data collection and variables

We tested our hypotheses using data from a variety of resources from the advanced imaging symposium and the 6 years of publication records from 2013 to 2018 on the attendees. In the analyses, we did not include the year 2012 to remove potential research topics or ideas that were in progress prior to the symposium. We used data from the scientists' registration form for the symposium, which contained information about their institution, department, academic position, self‐identity as an imager or clinician, and statements of interest. The sociometric badges automatically recorded their face‐to‐face encounters when two badges were facing each other with a direct line of sight within a 30° cone of 1 m. We verified that all recorded interactions were within 10 m using Bluetooth proximity data (Kim et al., 2012) and required that two badges be in contact with each other over a span of at least 1 min (Ingram & Morris, 2007). We collected face‐to‐face interaction data for 306 (74%) scientists who attended the symposium, and the subsequent analyses are based on these scientists.^3^ After the symposium, we collected information on the coapplicants and the awardees of the advanced imaging grants and used the Scopus database to collect scientists' publication records.

#### 3.3.1 Dependent variables

##### Knowledge transfer

We base our knowledge transfer measure on the MeSH lexicon. In the life sciences, the U.S. National Library of Medicine (NLM) uses a controlled MeSH taxonomy of keywords to index biomedical and health‐related information for articles appearing in MEDLINE/PubMed, the NLM Catalog, and other NLM databases. Each article is associated with a set of MeSH keywords that describe the content of the citation. MeSH keywords are assigned by professional science librarians and computer algorithms to ensure global and consistent assignment of keywords across the life sciences (Coletti & Bleich, 2001). We extracted the unique MeSH keywords associated with each scientist's publications to create two vectors of MeSH keywords: the first with all MeSH keywords prior to the advanced imaging symposium (i.e., pre‐2012), and the second with all MeSH keywords after the symposium (i.e., 2013–2018). For each scientist‐pair {*i*,*j*}, we then counted the number of MeSH terms that *j* transferred to *i* and the number of MeSH terms that *i* transferred to *j* by taking the intersection of MeSH keywords between *i*'s pre‐2012 vector and *j*'s post‐2012 vector and between *j*'s pre‐2012 vector and *i*'s post‐2012 vector, excluding any MeSH terms that were common to both *i* and *j* in their pre‐2012 MeSH vectors. Our resulting knowledge transfer measure, *MeSH keyword transfer* is the count of “transferred” MeSH keywords between pair {*i*,*j*}, normalized by the total number of MeSH keywords in scientists *i* and *j*'s post‐2012 MeSH vectors, expressed as a percentage. The resulting measure ranges from 0 to 100% to and is interpreted as the percentage of postsymposium MeSH keywords that were transferred between scientist‐pair {*i*,*j*}, with 0% representing no transferred MeSH keywords and 100% representing complete transfer.

##### Knowledge creation

We measure knowledge creation as the postsymposium (2013–2018) count of *Copublications* between scientist‐pair {*i*,*j*} in peer‐reviewed journals, conference proceedings, and book chapters.

##### Knowledge diffusion

We measure knowledge diffusion as the postsymposium (2013–2018) count of noncoauthored *Forward citations* between scientist‐pair {*i*,*j*} in peer‐reviewed journals, conference proceedings, and book chapters.

#### 3.3.2 Independent variables

##### Knowledge sharing

We use two alternative variables to capture knowledge sharing. First, we use the dummy variable, *Same room*, to measure whether scientist‐pair {*i*, *j*} was randomly assigned to the same room (treatment pairs) or different breakout rooms (control pairs). Second, we use the dummy variable, *F2F (face‐to‐face) communication* to measure whether scientist‐pair {*i*, *j*} engaged in least 1 min of interaction, recorded using sociometric badges.

##### Knowledge similarity

Knowledge similarity is comprised of field and intellectual similarity. We measure *Field similarity* using clinical areas (third‐party coded from the scientists' statements of interest), which pertain to the primary area of responsibility for “bedside” patient care. There were a total of 24 unique clinical areas (e.g., oncology, neurology, immunology), and some statements of interests (4.24%) spanned two clinical areas, such as neurology/endocrinology. We then use a categorical variable to indicate whether scientist‐pair {*i*, *j*} shared *Low*, *Moderate* or *High field similarity* depending on whether their clinical areas shared no (85.5%), partial (1.9%), or complete (12.6%) overlap, respectively.

We measure *Intellectual similarity* using the count of common MeSH keywords shared by scientist‐pair {*i*, *j*} prior to 2012 and dichotomize the distribution into three equal‐sized groups, to indicate whether scientist‐pair {*i*, *j*} had low, moderate, or a high number of common MeSH keywords prior to the 2012 symposium. *Low intellectual similarity* corresponds to 0–2 common keywords, *Moderate intellectual similarity* corresponds to 3–11 common keywords, and *High intellectual similarity* corresponds to more than 11 common keywords.

##### Elemental collaboration

We use *Grant coapplicant* to measure an elemental collaboration, which captured whether scientist‐pair {*i*, *j*} coapplied on the grant following the symposium.

#### 3.3.3 Other variables

The analysis strategy relies most critically on the research design's randomization. We use dummy variables for each symposium night and room (i.e., fixed effects) to control for unobserved night and room characteristics. To test Hypotheses (H2c) and (H2d), we use the dummy variable, *Grant awardee* to capture whether scientist‐pair {*i*,*j*} included a grant recipient. Among the 306 scientists, 13 pilot grant proposals were awarded funding, comprising 6.54% of pairs with grant awardees.

### 3.4 Estimation approach

We wish to estimate the effects of field and intellectual similarity between knowledge sharing partners on knowledge production outcomes. The unit of analysis is the scientist‐pair{*i*,*j*}, and pairs are considered to be “at risk” if they attended the same night of the symposium—a total of 15,817 pairs. First, we analyze the effect of being in the same (treatment) versus different (control) breakout rooms on knowledge production outcomes using ordinary least squares (OLS) regression. We then interact *Same room* with *Field similarity*, and *Same room* with *Intellectual similarity* to examine knowledge similarity effects. Second, we analyze the effect of face‐to‐face communication on knowledge production outcomes using instrumental variable (IV) regression. We use an IV approach to account for the common endogeneity issue in pairwise interaction data. We exploit exogenous variation in the likelihood of interaction between scientists who are assigned to the same versus different breakout rooms by using *Same room* as an instrument for *F2F communication* in the first stage, and the estimates for *F2F communication* in the second stage. We then interact *F2F communication* with *Field similarity*, and *F2F communication* with *Intellectual similarity* to examine knowledge similarity effects.

We address the nonindependence, common‐person problem of dyadic regressions by estimating robust *SE*s that are simultaneously clustered on both members of the dyad, using multiway clustering, developed theoretically by Cameron, Gelbach, and Miller (2011) and implemented for Stata in clus_nway.ado (Kleinbaum et al., 2013).

## 4 RESULTS

In this section, we begin by presenting descriptives of the main variables. Table 2 presents the means, standard deviations, and correlations of the main variables. We also note that the summary statistics of the covariates for same room versus different room pairs indicate that the randomization achieved balance across covariates (see Table A4, Supporting Information).

**TABLE 2:** Correlation between main variables (*N* = 15,817)

	Variable	Mean	*SD*	1	2	3	4	5	6	7
1	MeSH keyword transfer	3.693	3.709							
2	Copublications	0.031	1.004	0.010						
3	Forward citations	0.265	2.497	0.019	0.479					
4	Grant coapplicant	0.002	0.044	0.004	0.097	0.027				
5	Same room	0.266	0.442	0.024	−0.004	−0.001	0.006			
6	F2F communication	0.078	0.268	0.003	0.047	0.039	0.067	0.198		
7	Intellectual similarity	1.076	0.810	−0.005	0.037	0.043	0.037	−0.002	0.047	
8	Field similarity	0.272	0.673	0.437	0.029	0.074	0.017	−0.008	0.021	0.026

*Note*: Intellectual and field similarity are categorical variables with the following distributions: intellectual (low: 32.4%, moderate: 32.7%, high: 35.0%) and field (low: 85.5, 1.9, and 12.6%).

**FIGURE 2:**
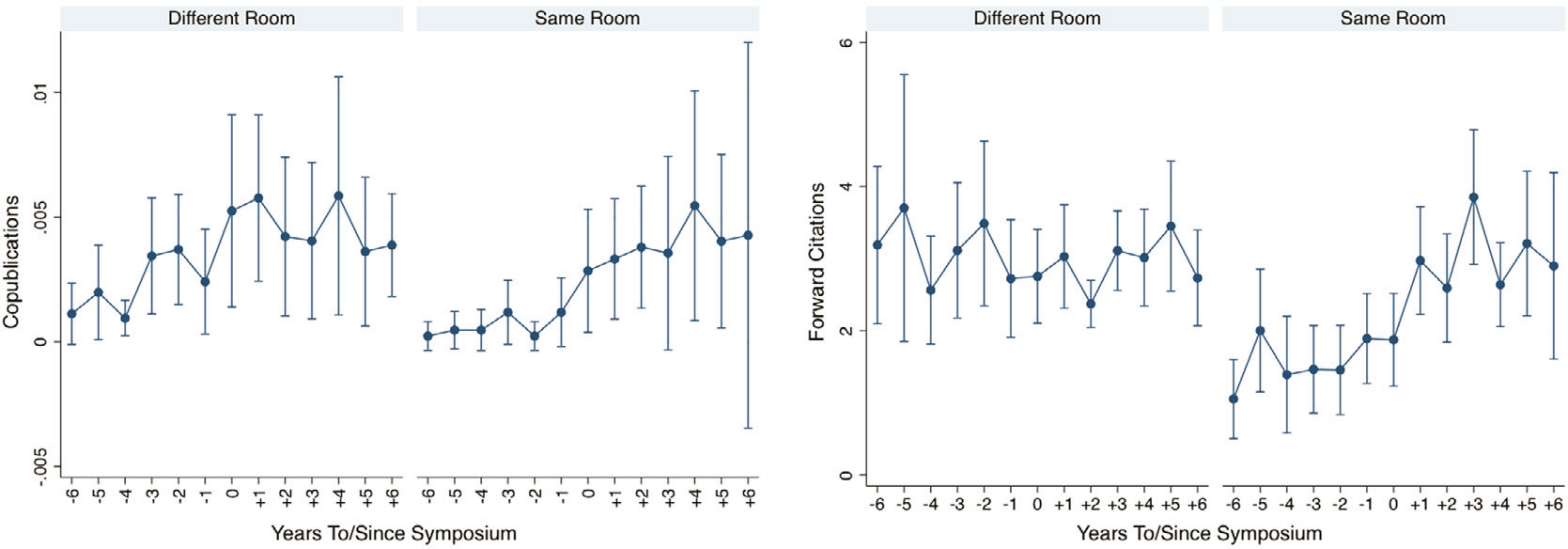
Yearly trend in copublication (left) and forward citation (right) rates between same room (treatment) and different room (control) pairs with 95% CIs

Figure 2 shows the yearly trend in copublication (left) and forward citation (right) rates between different room (*N* = 11,611) and same room (*N* = 4,206) pairs by year, in the 6 years to and since the symposium, with 95% CIs. The plots show that there were no differences between the same room and different room pairs before the knowledge sharing intervention. After the intervention, we observe an increase in copublication and citation rates for the same room pairs.

Next, we present our main regression results in subsections, beginning with knowledge transfer, then creation, and finally, diffusion. We note that the F‐statistics for the IV regressions are all above the threshold of 10 for strong instruments (Table A5, Supporting Information), and that our results are robust to the inclusion of all scientist‐pair covariates (Table A6, Supporting Information) and the reduced form (OLS) models for the full sample of participants (Tables A7–A9, Supporting Information).

### 4.1 Knowledge transfer results

We present the OLS and IV regression knowledge transfer results in Table 3. The dependent variable is the percentage of scientist‐pair {*i*,*j*}'s MeSH postsymposium keywords that were transferred between *i* and *j*, with 0% being no transfer, and 100% being complete transfer. Models 1 and 2 present the baseline models with main effects only; Models 3 and 4 add the field similarity interaction terms; Models 5 and 6 add the intellectual similarity interaction terms; and Models 7 and 8 show the full results with both interaction terms.

**TABLE 3:** Regression models of knowledge transfer—% of MeSH keywords transferred between scientist‐pair {*i*,*j*}; *N* = 15,817

Variables	Model 1 OLS	Model 2 IV	Model 3 OLS	Model 4 IV	Model 5 OLS	Model 6 IV	Model 7 OLS	Model 8 IV
Same room	0.107 (0.0979)		0.0344 (0.144)		0.0336 (0.138)		−0.0379 (0.172)	
F2F communication		0.838 (0.763)		0.214 (1.108)		0.233 (1.025)		−0.512 (1.352)
Same room × low field similarity			0.0707 (0.150)				0.0686 (0.150)	
Same room × moderate field similarity			0.712 (0.471)				0.681 (0.468)	
Same room × low intellectual similarity					−0.175 (0.218)		−0.171 (0.218)	
Same room × moderate intellectual similarity					0.362 (0.177)		0.361 (0.177)	
F2F × low field similarity				0.621 (1.204)				0.804 (1.239)
F2F × moderate field similarity				4.088 (3.169)				4.059 (3.379)
F2F × low intellectual similarity						−1.459 (1.753)		−1.531 (1.869)
F2F × moderate intellectual similarity						3.348 (1.564)		3.305 (1.644)
Low field similarity	0.140 (0.135)	0.171 (0.135)	0.121 (0.143)	0.104 (0.183)	0.141 (0.135)	0.176 (0.137)	0.123 (0.143)	0.0900 (0.189)
Moderate field similarity	−0.130 (0.274)	−0.136 (0.264)	−0.316 (0.269)	−0.606 (0.381)	−0.136 (0.272)	−0.174 (0.274)	−0.315 (0.268)	−0.638 (0.398)
Low intellectual similarity	−4.130 (0.238)	−4.120 (0.237)	−4.130 (0.238)	−4.123 (0.238)	−4.081 (0.246)	−4.015 (0.282)	−4.082 (0.246)	−4.013 (0.289)
Moderate intellectual similarity	−0.880 (0.153)	−0.862 (0.152)	−0.880 (0.153)	−0.866 (0.152)	−0.974 (0.163)	−1.095 (0.196)	−0.974 (0.163)	−1.096 (0.204)
Night FE	Y	Y	Y	Y	Y	Y	Y	Y
Room FE	Y	Y	Y	Y	Y	Y	Y	Y
*R*‐squared	.224	.219	.224	.218	.224	.201	.224	.216

*Note*: Multiway, robust *SE*s in parentheses. Significance stars (*) are omitted.

Hypotheses (H1a) and (H1b) suggest that field and intellectual similarity have an inverted U‐shaped effect on the relationship between knowledge sharing and knowledge transfer, respectively. Models 3 and 4 indicate that there is no meaningful association between moderate field similarity and knowledge transfer among same room pairs (0.712, *p* = .132) and communicating pairs (4.088, *p* = .197). Models 5 and 6 show that compared to pairs with high intellectual overlap, scientists with moderate intellectual similarity transferred a higher percentage of MeSH keywords for both same room pairs (0.362, *p* = .042) and communicating pairs (3.348, *p* = .032), respectively. These results are consistent in Models 7 and 8, which include both interaction terms.

**FIGURE 3:**
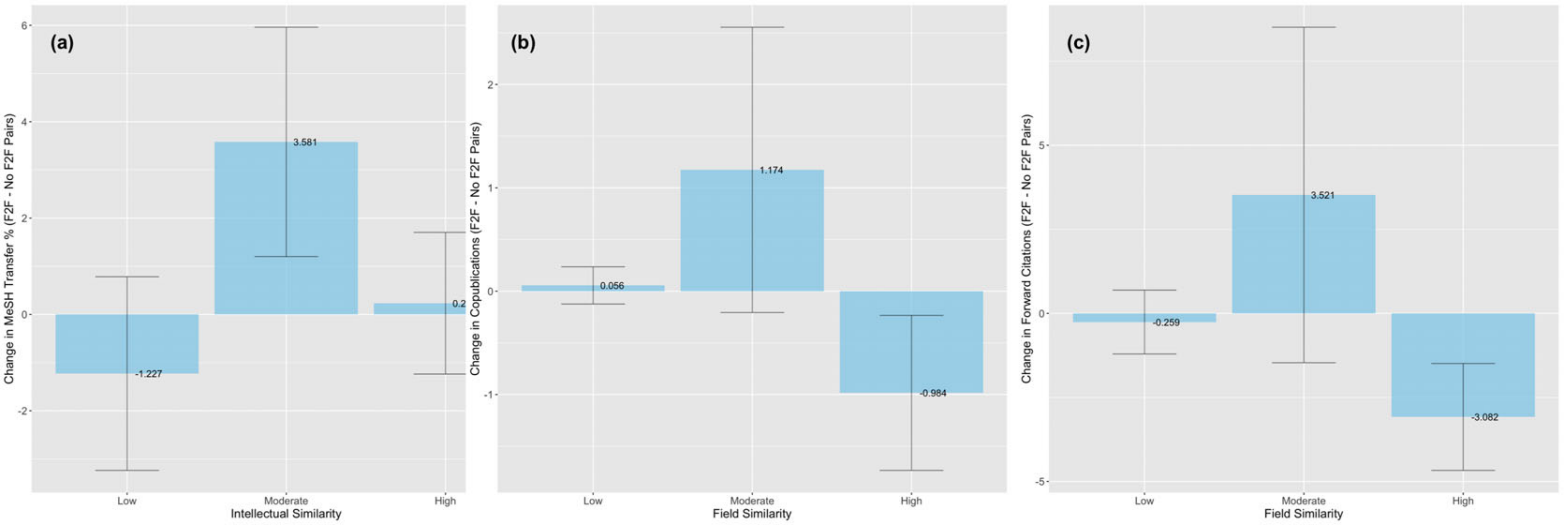
Change in (a) knowledge transfer, (b) knowledge creation, and (c) knowledge diffusion and knowledge similarity for communicating and noncommunicating pairs

Figure 3a shows the change in knowledge transfer among communicating versus noncommunicating pairs and intellectual similarity from Model 4, with 95% CIs, and illustrates the inverted U‐shaped relationship. While communication did not meaningfully impact knowledge transfer among pairs with low field overlap, it led to an increase of 3.581% among pairs with moderate intellectual overlap from 4.085% [3.795%, 4.376%] to 7.666% [4.995%, 10.338%] percent, a nearly twofold increase, compared to a 0.233% [−1.239%, 1.705%] increase for high intellectual overlap pairs. The percentage increase among moderately overlapping pairs corresponds to about six new MeSH keywords per scientist, roughly equivalent to the average number of MeSH keywords in one publication.^4^ This result supports Hypothesis (H1b) by showing cooperative effects of knowledge transfer for pairs with moderate intellectual similarity.

### 4.2 Knowledge creation

We present the OLS and IV knowledge creation regression results in Table 4. The dependent variable is the number of copublications between scientist‐pair {*i*,*j*}. Models 1 and 2 present the baseline models with main effects only; Models 3 and 4 add the field similarity interaction terms; Models 5 and 6 add the intellectual similarity interaction terms; Models 7 and 8 show the full results with both interaction terms; Model 9–11 adds *Grant coapplicant*, followed by the field and intellectual similarity interaction terms, as well as both interaction terms, and controls for *Grant awardee*.

**TABLE 4:** Regression models of knowledge creation—# of copublications between scientist‐pair {*i*,*j*}; *N* = 15,817

Variables	Model 1 OLS	Model 2 IV	Model 3 OLS	Model 4 IV	Model 5 OLS	Model 6 IV	Model 7 OLS	Model 8 IV	Model 9 OLS	Model 10 OLS	Model 11 OLS
Same room	−0.00683 (0.0144)		−0.127 (0.0769)		−0.0294 (0.0360)		−0.148 (0.0931)		−0.00784 (0.0148)	−0.00822 (0.0148)	−0.00799 (0.0147)
F2F communication		−0.0535 (0.111)		−0.984 (0.616)		−0.221 (0.268)		−1.147 (0.746)			
Same room × low field sim.			0.133 (0.0788)				0.132 (0.0783)				
Same room × mod. field sim.			0.329 (0.165)				0.330 (0.164)				
Same room × low intellectual sim.					0.0306 (0.0378)		0.0307 (0.0372)				
Same room × mod. intellectual sim.					0.0409 (0.0361)		0.0397 (0.0355)				
F2F × low field sim.				1.040 (0.634)				1.048 (0.637)			
F2F × mod. field sim.				2.159 (1.029)				2.110 (1.022)			
F2F × low intellectual sim.						0.240 (0.297)		0.205 (0.306)			
F2F × mod. intellectual sim.						0.333 (0.280)		0.332 (0.290)			
Grant coapplicant									3.708 (1.583)	2.482 (1.211)	3.810 (1.614)
Coapplicant × low field sim.									−2.427 (1.845)		−2.299 (2.109)
Coapplicant × mod. field Sim.									−3.303 (1.621)		−3.405 (1.659)
Coapplicant × low intellectual sim.										−0.639 (2.085)	−0.324 (2.236)
Coapplicant × mod. intellectual sim.										−1.083 (1.482)	−0.571 (1.632)
Awardee									0.000295 (0.0140)	−0.00167 (0.0137)	−2.47e‐05 (0.0140)
Low field sim.	−0.109 (0.0540)	−0.111 (0.0566)	−0.14 (0.0735)	−0.220 (0.120)	−0.109 (0.0539)	−0.112 (0.0579)	−0.144 (0.0733)	−0.222 (0.121)	0.0552 (0.0422)	0.0405 (0.0441)	0.0552 (0.0422)
Moderate field sim.	−0.0529 (0.0659)	−0.0525 (0.0655)	−0.139 (0.0701)	−0.297 (0.132)	−0.0534 (0.0660)	−0.0567 (0.0675)	−0.140 (0.0706)	−0.296 (0.130)	0.0887 (0.0536)	0.0981 (0.0539)	0.0887 (0.0536)
Low intellectual sim.	−0.0696 (0.0222)	−0.0702 (0.0226)	−0.0695 (0.0221)	−0.0738 (0.0240)	−0.0777 (0.0297)	−0.0908 (0.0437)	−0.0777 (0.0294)	−0.0916 (0.0455)	−0.0651 (0.0222)	−0.0643 (0.0224)	−0.0645 (0.0224)
Moderate intellectual sim.	−0.0657 (0.0209)	−0.0669 (0.0219)	−0.0656 (0.0207)	−0.0699 (0.0231)	−0.0765 (0.0272)	−0.0928 (0.0399)	−0.0760 (0.0268)	−0.0954 (0.0418)	−0.0602 (0.0209)	−0.0591 (0.0209)	−0.0593 (0.0210)
Night FE	Y	Y	Y	Y	Y	Y	Y	Y	Y	Y	Y
Room FE	Y	Y	Y	Y	Y	Y	Y	Y	Y	Y	Y
*R*‐squared	.003	.002	.004	—	.003	—	.004	.012	.015	.012	.015

*Note*: Multiway robust *SE*s in parentheses. Significance stars (*) are omitted.

Hypotheses (H2a) and (H2b) predict that field and intellectual similarity have an inverted U‐shaped effect on the relationship between knowledge sharing and knowledge creation, respectively, while Hypotheses (H2c) and (H2d) predict that knowledge sharing partners who start an elemental collaboration are more likely to copublish when they share greater field and intellectual overlap, respectively. Models 3 and 4 show that compared to same field pairs, moderate field similarity has a strong positive relationship with knowledge creation among same room pairs (0.329, *p* = .047) and communicating pairs (2.159, *p* = .036). There is also a smaller, positive association between low field similarity and knowledge creation among same room pairs (0.133, *p* = .092) and communicating pairs (1.040, *p* = .101). Turning to intellectual similarity, Models 5 and 6 show there is no meaningful association between moderate intellectual similarity and knowledge creation among same room pairs (0.0409, *p* = .257) and communicating pairs (0.333, *p =* .234). The results are consistent in Models 7 and 8, which includes both interaction terms.

Figure 3b plots the change in copublications between communicating and noncommunicating pairs and field similarity, with 95% CIs from Model 3, and illustrates the estimated inverted U‐shaped relationship. We observe that while communication did not meaningfully benefit pairs with low field overlap, communication led to an increase of 1.174 [−0.207, 2.556] copublications among pairs with moderate field overlap, and −0.984 [−1.731, −0.237] fewer copublications among pairs with high field overlap. The patterns suggest that communication reduced the search costs of identifying synergistic collaborations with scientists sharing some field interests, resulting in a reallocation of resources away from pairs with high field overlap.

We also examined the extent that the knowledge sharing intervention impacted the scientists' overall research portfolios. Turning to the scientists' postsymposium publications, each scientist had an average of *M* = 32.394 (*SD* = 35.412) peer‐reviewed research articles; the increase of 1.174 publications among pairs with moderate field overlap corresponds to about 7% of a scientist's publications, which is of considerable magnitude from a 90 min intervention. Turning to changes in research direction, we also note that same room pairs were more likely to publish in advanced imaging journals (e.g., *Magnetic Resonance in Medicine*, *NMR in Biomedicine*): 21% for same room versus 6% for different room pairs (*t* = 2.302, *p* = .0249).^5^ This suggests that the knowledge sharing treatment not only reduced search costs of finding collaborators but also potentially reshaped the pairs' research trajectories.

Turning to Hypothesis (H2c), Model 9 shows that controlling for *Grant awardee*, grant coapplicants are more likely to copublish when they are from the same field (low: −2.427, *p* = .189; moderate: −3.303, *p* = .042). Finally, turning to H2d, Model 10 indicates that intellectual similarity is not a meaningful predictor of elemental tie persistence (low: −0.639, *p* = .759; moderate: −1.083, *p* = .465). The results remain consistent in Model 11, which includes both interaction terms. This suggests that although strong field overlap is a catalyzing force for initiating early‐stage collaborations, these requirements may dissipate over the long‐run as these ties persist into copublications. Thus, our results show support for Hypotheses (H2a) and (H2c).

### 4.3 Knowledge diffusion

We present the OLS and IV knowledge diffusion regression results in Table 5. The dependent variable is the number of forward citations between scientist‐pair {*i*,*j*}. Models 1 and 2 present the baseline models with main effects only; Models 3 and 4 add the field similarity interaction terms; Models 5 and 6 add the intellectual similarity interaction terms; Models 7 and 8 show the full results with both interaction terms.

**TABLE 5:** Regression models of knowledge diffusion—# of forward citations between scientist‐pair {*i*,*j*}; *N* = 15,817

Variables	Model 1 OLS	Model 2 IV	Model 3 OLS	Model 4 IV	Model 5 OLS	Model 6 IV	Model 7OLS	Model 8 IV
Same room	−0.0687 (0.0710)		−0.399 (0.165)		−0.0540 (0.150)		−0.384 (0.211)	
F2F communication		−0.537 (0.551)		−3.082 (1.331)		−0.391 (1.115)		−2.910 (1.656)
Same room × low field similarity			0.366 (0.162)				0.367 (0.162)	
Same room × moderate field similarity			1.003 (0.605)				0.999 (0.605)	
Same room × low intellectual similarity					−0.0487 (0.146)		−0.0476 (0.146)	
Same room × low intellectual similarity					−0.00251 (0.145)		−0.00592 (0.145)	
F2F × low field similarity				2.823 (1.324)				2.835 (1.332)
F2F × moderate field similarity				6.603 (3.364)				6.679 (3.334)
F2F × low intellectual similarity						−0.426 (1.154)		−0.537 (1.162)
F2F × moderate intellectual similarity						−0.0908 (1.138)		−0.112 (1.135)
Low field similarity	−0.316 (0.129)	−0.336 (0.137)	−0.413 (0.165)	−0.633 (0.256)	−0.316 (0.129)	−0.335 (0.140)	−0.413 (0.165)	−0.632 (0.258)
Moderate field similarity	−0.209 (0.208)	−0.205 (0.214)	−0.471 (0.185)	−0.955 (0.360)	−0.209 (0.208)	−0.204 (0.215)	−0.471 (0.186)	−0.962 (0.358)
Low intellectual similarity	−0.446 (0.0920)	−0.452 (0.0926)	−0.446 (0.0918)	−0.462 (0.0950)	−0.433 (0.102)	−0.417 (0.141)	−0.433 (0.102)	−0.418 (0.143)
Moderate intellectual similarity	−0.353 (0.0762)	−0.364 (0.0791)	−0.352 (0.0760)	−0.373 (0.0810)	−0.352 (0.0866)	−0.354 (0.128)	−0.351 (0.0862)	−0.361 (0.130)
Night FE	Y	Y	Y	Y	Y	Y	Y	Y
Room FE	Y	Y	Y	Y	Y	Y	Y	Y
*R*‐squared	.011	.004	.011	—	.011	.004	.011	—

*Note*: Multiway, robust *SE*s in parentheses. Significance stars (*) are omitted.

Hypotheses (H3a) and (H3b) predict that knowledge sharing partners are less likely to diffuse knowledge as their field and intellectual similarity increase, respectively. Models 3 and 4 show that increasing field similarity reduced knowledge diffusion among same room pairs (low: 0.366, *p* = .024; moderate: 1.003, *p* = .098) and communicating pairs (low: 2.823, *p* = .033; moderate: 6.603, *p* = .050). Models 5 and 6 show that there is no evidence that high intellectual similarity lowered knowledge diffusion among same room (low: −0.0487, *p* = .739; moderate: −0.003, *p* = .986) or communicating pairs (low: −0.426, *p* = .712; moderate: −0.091, *p* = .936). The results are consistent in Models 7 and 8, which include both knowledge similarity interaction terms.

Figure 3c plots the change in knowledge diffusion (forward citations) between communicating and noncommunicating pairs from Model 4, with 95% CIs. We observe that while communication did alter forward citation trends for pairs with low field overlap, and led to a small increase of 3.521 [−1.465, 8.508] citations for pairs with moderate field overlap, we observe a large decrease from 0.874 [0.347, 1.402] to −2.207 [−4.326, −0.088] for same field pairs, corresponding to a reduction of about 3.082 citations per pair.

To generate greater insight into the types of publications being cited among the low and moderate field overlap pairs, we compared the publication titles of cited papers for pairs assigned to the same versus different rooms. We observe that the same room pairs were more likely to cite papers using advanced imaging technologies (e.g., sample paper titles included: “Massively parallel MRI detector arrays”; “An fMRI study of facial emotion processing patients with schizophrenia”). Overall, there was a greater use of advanced imaging technologies in the publication titles of same room pairs: 42.6% for same room pairs versus 31.3% for different room pairs (*t* = 2.406, *p* = .017).^6^ This suggests that the knowledge sharing intervention enabled pairs from different fields to learn about “outside” work, which they subsequently integrated into their own future research. In contrast, the knowledge sharing intervention may have led to a crowding out effect among highly similar pairs. In summary, we find evidence for competitive effects of field similarity on knowledge diffusion, which confirms Hypothesis (H3a).

## 5 DISCUSSION

The ability to produce and manage knowledge is a critical source of competitive advantage for organizations of all types (Argote, 2012; Hargadon & Sutton, 1997). Yet the knowledge production process is both time‐intensive and uncertain (Maggitti, Smith, & Katila, 2013). To address this issue, the premise of this article is based on the notion that “engineering serendipity” can promote greater knowledge sharing and more efficient knowledge production. We systematically examine how the degree of field and intellectual similarity between knowledge sharing partners affects the likelihood that engineered encounters affects three knowledge production outcomes: knowledge transfer, creation, and diffusion.

We find both cooperative and competitive effects of serendipitous knowledge sharing on knowledge production. On the one hand, knowledge sharing leads to greater knowledge transfer and creation when people share some overlapping research interests: knowledge sharing partners are more likely to transfer and acquire MeSH keywords from one another when they already have some intellectual overlap and more likely to copublish when they share field overlap, with field similarity being more critical to collaborations in the near‐term than over longer horizons. On the other hand, knowledge sharing appears to have little benefit for pairs with low knowledge similarity and reduces knowledge diffusion between people from the same field: we observe that pairs from the same field are less likely to cite each other after interacting.

These findings make a number of contributions to the literature. First, we contribute to the literature on search costs and information frictions associated with knowledge production (Agrawal & Goldfarb, 2008; Boudreau et al., 2017; Catalini, 2018; Hansen, 1999; Szulanski, 1996), by focusing on the extent that “engineered serendipity” can mitigate some of these barriers. Engineered, serendipitous encounters differ from traditional knowledge production mechanisms because they give people a common time and space to meet, thereby removing some of the search costs associated with finding suitable knowledge sharing partners. Our work suggests that engineered serendipity creates opportunities for synergistic collaborations over the long‐run that have the potential to broaden collaboration networks and reshape research trajectories. Consistent with the information frictions explanation, knowledge overlap is a greater constraint on near‐term than long‐term knowledge creation. Knowledge similarity can be a catalyzing force for near‐term research activities because there are fewer start‐up costs and uncertainties associated with initiating collaborations with similar others, such as people working in the same field. In Boudreau et al. (2017), we found that the knowledge sharing intervention's effect on grant coapplications was most beneficial for pairs from the same field. This suggests that in the short‐term, opportunity and discretion remain important factors driving knowledge production (Dahlander & McFarland, 2013). We expand on these findings to show that while field overlap remains a difficult hurdle to overcome in the near‐term, over time, scientists place smaller premiums on knowledge similarity, instead emphasizing synergistic collaborations where partners have both common knowledge and divergent expertise. This shift may be due to the time and repeated interactions needed to develop an initial encounter into a multiplex relationship (Ingram & Morris, 2007; Uzzi, 1997), or the evolving expectations from collaborators as projects move from exploratory research to joint execution of research ideas. They also suggest that homophilous collaborations (Dahlander & McFarland, 2013) can be attributed in part to search costs and information costs.

Second, this study advances understanding of how opportunities for temporary colocation, offered by conferences, symposia and similar events potentially impact the direction and quality of scientific knowledge production. Although prior research suggests that temporary colocation is critical to knowledge production (Campos et al., 2018; Catalini, 2018; Catalini et al., 2020; Chai & Freeman, 2019), it has typically inferred face‐to‐face interaction from observational data, rather than actual acts of knowledge sharing. We suggest that one attractive aspect of conferences over informal serendipitous encounters (e.g., in hallways, watercoolers) is that they have a focusing effect on the topic of knowledge sharing and idea exchange. This may be why even brief, highly structured, information‐rich interventions can have long‐term consequences on knowledge production, most notably by promoting research activities on the conference's topics of inquiry. It is important to note that the effects of these events go beyond monetary incentives, which may be an early catalyst to attract participants but the effects are more widespread and longer lasting.

Third, we make empirical and methodological contributions by designing a prospective study that amalgamates multiple sources and uses of data, namely a natural field experiment, direct observations of face‐to‐face communications and archival publication data to study long‐run knowledge production outcomes. This careful combination of data sources enables us to investigate the causal relationships between knowledge sharing and knowledge production over different time horizons, which are not possible with archival data (Fleming et al., 2007). Given the rise in field experiments in innovation research (Chatterji, Delecourt, Hasan, & Koning, 2019), research designs that at the outset plan for short‐ and long‐term time horizons may yield deeper insights that can help to offset the costs associated with multiple forms of data collection. In addition, our research design can be used as a template for future experiments with multiterm horizons, in terms of learning from its features and its potential shortcomings.

In this research, we have made a thorough effort to analyze how knowledge sharing leads to knowledge production in science. However, our study has some empirical limitations. First, our study focused on an interdisciplinary setting in a large, highly selective university. Therefore, it may be interpreted as a best‐case scenario in fostering knowledge production, as we must draw a boundary around the network and incentives under consideration. The symposium was highly structured to facilitate knowledge sharing, while knowledge production is a core activity for academics. Second, the geographic proximity of scientists may have facilitated scheduled or serendipitous encounters with greater ease (Catalini, 2018). Third, our focus on the life sciences also draws upon a specific population, where intragroup competition is normative (Haas & Park, 2010), in part due to the “winner‐takes‐all” model of rewards and recognition (Stephan, 1996). We recognize that other settings may feature greater geographic dispersion or promote more openness and cooperation; a promising future direction would be to extend this research to different settings.

We also focused most heavily on archival data (e.g., public trace data from publications) to examine long‐term knowledge production outcomes, which provided greater insights into the scientists' behaviors rather than the reasons behind the observed patterns. Although we made efforts to qualify these findings by examining the content of the knowledge transfer, publications, and citations, future research can seek to supplement archival data with alternative methods (e.g., surveys, interviews) that continue to track the scientists' interaction patterns, as well as the invisible track of “failed” knowledge production. Such complementary data sources will ultimately provide deeper insights on how engineered serendipitous encounters between people can be cultivated into productive relationships over time. This future work would facilitate a deeper performative assessment of how knowledge sharing can be “serendipitously engineered” to shape the quality of knowledge outcomes.

Overall, this study takes a critical step towards identifying the processes that explain when serendipitous encounters shape knowledge production outcomes among innovating individuals. We show that brief, information‐rich interactions between people with some overlapping knowledge interests can have a productive effect on knowledge transfer, creation and diffusion.

## Supporting information


**Appendix S1:** Supporting information

## Footnotes

^1^

The same field experiment was used in Boudreau et al. (2017) to investigate the effect of search costs on collaborations in the immediate aftermath of the experiment. It did not include long‐run knowledge production outcomes or leverage the sociometric badge data, described in Section 3.3.

^2^

Compared to the entire Harvard Medical School (HMS) population (*N* = 22,625), participants were more likely to be PhDs, had more prior publications on average and were more likely to be instructors, assistant or associate professors relative to the overall distribution at HMS. These differences are consistent with the academic research setting of the symposium and the pilot grant funding opportunity. See Table A1 for summary statistics between participants and the HMS population.

^3^

Badges were randomly assigned to all symposium attendees. 26% of badges malfunctioned and did not record face‐to‐face interaction data; participants were not aware of whether they were assigned a working or faulty badge. For each symposium night, the sociometric badges recorded interactions for 64.4, 68.2, and 90.4% of the participants (Table A2). We conducted balance checks on all available scientist covariates between the complete and observed sample and found no differences between the samples (Table A3). As robustness, we also perform the main OLS regression analyses for each knowledge production outcome on the full sample of participants to verify that the results are directionally consistent across the full sample and the observed badge sample.

^4^

In the postsymposium period, each scientist has *M* = 172.57 (*SD* = 142.61) MeSH keywords and *M* = 6.85 (*SD* = 4.28) MeSH keywords per publication.

^5^

A journal was coded as an advanced imaging if the journal title included any of the three technologies featured at the symposium (physiological magnetic resonance, positron emission tomography, and optical imaging). There were 48 unique journals for same room versus 169 unique journals for the different room pairs.

^6^

There were a total of 149 (same room) and 415 (different room) forward citations for pairs with low/moderate field overlap, respectively. A publication title was coded as advanced imaging if it included any of the three technologies featured at the symposium (physiological magnetic resonance, positron emission tomography, and optical imaging).
