# Supplementary material for: Engineering serendipity: When does knowledge sharing lead to knowledge production?
Source: Strateg Manag J. 2020 Nov 24;42(6):1215–44. doi: 10.1002/smj.3256 (PMC8297436; doi:10.1002/smj.3256)
Supplement: Supplementary file 1 — Appendix S1: Supporting information [file SMJ-42-1215-s001.docx]

**APPENDIX for**

***Engineering Serendipity: When Does Knowledge Sharing Lead to Knowledge Production?***

Table A1. Summary Statistics of HMS Population vs. Symposium Participants

| Sample Means | *HMS* | *Participants* | *Difference* |
| --- | --- | --- | --- |
|  | *Profiles* |  |  |
| Degree |  |  |  |
| MD | 0.604 | 0.572 | 0.031 |
| PhD | 0.382 | 0.493 | -0.111 |
| Publications | 17.837 | 22.169 | -4.332 |
| Rank |  |  |  |
| Professor | 0.061 | 0.037 | 0.024 |
| Associate Professor | 0.066 | 0.157 | -0.091 |
| Assistant Professor | 0.108 | 0.204 | -0.096 |
| Instructor | 0.278 | 0.331 | -0.052 |
| Postdoc/Fellow | 0.401 | 0.219 | 0.182 |
| Other | 0.086 | 0.052 | 0.034 |
| Longwood | 0.619 | 0.510 | 0.109 |
| Hospital |  |  |  |
| Beth Israel Deaconess Medical Center | 0.128 | 0.139 | -0.011 |
| Massachusetts General | 0.245 | 0.371 | -0.125 |
| Brigham and Women's | 0.201 | 0.184 | 0.017 |
| Children's Hospital | 0.120 | 0.129 | -0.009 |
| Radiology Department | 0.054 | 0.266 | -0.213 |
| Observations | 22,625 | 402 |  |

Table A2. Comparison of Observed and Full Sample of Participants by Night, Room and Group

| Symposium Night | Count (%) of Total |
| --- | --- |
| 1 | 92 (63.4%) |
| 2 | 90 (68.2%) |
| 3 | 124 (90.4%) |
| Total | 306 (73.9%) |
| Breakout Room | Count (%) of Total |
| 1 | 57 (60.6%) |
| 2 | 114 (85.7%) |
| 3 | 63 (67.0%) |
| 4 | 72 (77.2%) |
| Total | 306 (73.8%) |
| Poster Group | Count (%) of Total |
| 1 | 150 (73.9%) |
| 2 | 156 (73.8%) |
| Total | 306 (73.8%) |

Note: N = 413 observations for the full sample, comprised of 402 participants (392 scientists that attended one night of the symposium and 10 “super experts” with deep expertise in advanced imaging technologies who were invited to attend between one and three symposium nights, for a total of 21 observations).

Table A3. Covariate Balance Checks Between Observed and Full Sample of Participants

| Covariate | χ^2^ test statistic and p value |
| --- | --- |
| Female | χ^2^ (1) = 0.1159, p = 0.734 |
| Faculty rank | χ^2^ (7) = 5.6566, p = 0.580 |
| Institution | χ^2^ (23) = 24.076, p = 0.400 |
| Department | χ^2^ (55) = 47.579, p = 0.751 |
| Clinical area | χ^2^ (38) = 45.223, p = 0.196 |
| Imager | χ^2^ (1) = 0.4540, p = 0.500 |
| Imaging technology | χ^2^ (19) = 20.167, p = 0.385 |
| Super expert | χ^2^ (1) = 5.281, p = 0.022 |

Note: N = 413 observations for the full sample, comprised of 402 participants (392 scientists attending one night of the symposium and 10 “super experts” with deep expertise in advanced imaging technologies who were invited to attend between one and three symposium nights, for a total of 21 observations).

Table A4. Summary Statistics For Same Room versus Different Room Pairs (N = 15,817)

| Covariate | Treatment:  Same Room | Control:  Different Room | Difference |
| --- | --- | --- | --- |
| Previous coauthor | 0.001 | 0.002 | -0.001 |
| Same institution | 0.179 | 0.189 | -0.010 |
| Same department | 0.102 | 0.097 | 0.005 |
| Same imaging tech. | 0.175 | 0.171 | 0.003 |
| Both imagers | 0.170 | 0.175 | -0.005 |
| Both clinicians | 0.343 | 0.335 | 0.009 |
| Same rank | 0.202 | 0.218 | -0.016 |
| Both female | 0.084 | 0.084 | -0.000 |
| Both male | 0.506 | 0.496 | 0.001 |

Table A5. First Stage OLS Regression Models of F2F Communication on Same Room, Field Similarity and Intellectual Similarity (N = 15,817)

| VARIABLES | Model 1 | Model 2 | Model 3 | Model 4 |
| --- | --- | --- | --- | --- |
| Same room | 0.128 | 0.137 | 0.136 | 0.145 |
|  | (0.00888) | (0.0211) | (0.0129) | (0.0230) |
| Same room x Low field sim. |  | -0.0111 |  | -0.0108 |
|  |  | (0.0225) |  | (0.0226) |
| Same room x Mod. field sim. |  | 0.0420 |  | 0.0424 |
|  |  | (0.0522) |  | (0.0525) |
| Same room x Low intellectual sim. |  |  | -0.00673 | -0.00619 |
|  |  |  | (0.0170) | (0.0171) |
| Same room x Mod. intellectual sim. |  |  | -0.0196 | -0.0194 |
|  |  |  | (0.0166) | (0.0166) |
| Low field sim. | -0.0374 | -0.0344 | -0.0375 | -0.0346 |
|  | (0.00923) | (0.00841) | (0.00923) | (0.00841) |
| Moderate field sim. | 0.00704 | -0.00392 | 0.00729 | -0.00376 |
|  | (0.0293) | (0.0246) | (0.0293) | (0.0247) |
| Low intellectual sim. | -0.0114 | -0.0114 | -0.00961 | -0.00979 |
|  | (0.00706) | (0.00705) | (0.00591) | (0.00589) |
| Moderate intellectual sim. | -0.0214 | -0.0215 | -0.0163 | -0.0164 |
|  | (0.00627) | (0.00627) | (0.00566) | (0.00565) |
| Night FE | Y | Y | Y | Y |
| Room FE | Y | Y | Y | Y |
| First stage F-statistic | 361.031 | 173.583 | 184.852 | 103.413 |
| R-squared | 0.045 | 0.045 | 0.045 | 0.046 |

Multi-way, robust standard errors in parentheses.

Table A6. Regression Models of Knowledge Outcomes With Pairwise Covariates; N = 15,817

| VARIABLES | Model 1  Transfer  OLS | Model 2  Transfer  IV | Model 3  Creation  OLS | Model 4  Creation  IV | Model 5  Creation  OLS | Model 6  Diffusion  OLS | Model 7  Diffusion  IV |
| --- | --- | --- | --- | --- | --- | --- | --- |
| Same room | -0.0694 |  | -0.0901 |  | 0.00193 | -0.262 |  |
|  | (0.166) |  | (0.0698) |  | (0.0133) | (0.173) |  |
| F2F communication |  | -0.808 |  | -0.698 |  |  | -1.959 |
|  |  | (1.284) |  | (0.544) |  |  | (1.301) |
| Same room x Low field sim. | 0.0961 |  | 0.0883 |  |  | 0.276 |  |
|  | (0.146) |  | (0.0596) |  |  | (0.128) |  |
| Same room x Mod. field sim. | 0.670 |  | 0.273 |  |  | 0.860 |  |
|  | (0.482) |  | (0.149) |  |  | (0.569) |  |
| Same room x Low intellectual sim. | -0.149 |  | 0.0132 |  |  | -0.0795 |  |
|  | (0.211) |  | (0.0311) |  |  | (0.136) |  |
| Same room x Mod. intellectual sim. | 0.395 |  | 0.0246 |  |  | -0.0356 |  |
|  | (0.173) |  | (0.0316) |  |  | (0.137) |  |
| F2F x Low field similarity |  | 1.064 |  | 0.700 |  |  | 2.110 |
|  |  | (1.204) |  | (0.475) |  |  | (1.036) |
| F2F x Mod. field sim. |  | 3.999 |  | 1.715 |  |  | 5.697 |
|  |  | (3.463) |  | (0.893) |  |  | (3.046) |
| F2F x Low intellectual sim. |  | -1.330 |  | 0.0704 |  |  | -0.778 |
|  |  | (1.795) |  | (0.247) |  |  | (1.066) |
| F2F x Mod. intellectual sim. |  | 3.629 |  | 0.210 |  |  | -0.353 |
|  |  | (1.628) |  | (0.251) |  |  | (1.056) |
| Grant coapplicant |  |  |  |  | 2.989 |  |  |
|  |  |  |  |  | (1.274) |  |  |
| Coapplicant x Low field sim. |  |  |  |  | -2.913 |  |  |
|  |  |  |  |  | (1.705) |  |  |
| Coapplicant x Mod. field sim. |  |  |  |  | -2.635 |  |  |
|  |  |  |  |  | (1.311) |  |  |
| Coapplicant x Low intellectual sim. |  |  |  |  | 0.901 |  |  |
|  |  |  |  |  | (2.116) |  |  |
| Coapplicant x Mod. field sim. |  |  |  |  | 0.696 |  |  |
|  |  |  |  |  | (1.415) |  |  |
| Low field sim. | -0.403 | -0.668 | -0.00190 | -0.0782 | 0.0455 | -0.0671 | -0.329 |
|  | (0.222) | (0.375) | (0.0113) | (0.0623) | (0.0406) | (0.0751) | (0.223) |
| Moderate field sim. | -0.109 | -0.0510 | 0.0932 | 0.142 | 0.0529 | 0.293 | 0.447 |
|  | (0.142) | (0.189) | (0.0530) | (0.0860) | (0.0389) | (0.134) | (0.198) |
| Low intellectual sim. | -4.044 | -3.989 | -0.0331 | -0.0378 | -0.0290 | -0.316 | -0.282 |
|  | (0.244) | (0.283) | (0.0128) | (0.0237) | (0.00960) | (0.0752) | (0.106) |
| Moderate intellectual sim. | -0.973 | -1.113 | -0.0372 | -0.0478 | -0.0284 | -0.254 | -0.244 |
|  | (0.162) | (0.203) | (0.0130) | (0.0235) | (0.0111) | (0.0639) | (0.0997) |
| Previous coauthor | -0.302 | -0.526 | 6.274 | 6.311 | 6.181 | 12.72 | 12.84 |
|  | (0.903) | (1.001) | (3.511) | (3.550) | (3.581) | (6.666) | (6.722) |
| Same institution | -0.0145 | -0.0338 | 0.0691 | 0.0704 | 0.0657 | 0.127 | 0.141 |
|  | (0.107) | (0.111) | (0.0258) | (0.0275) | (0.0257) | (0.0588) | (0.0657) |
| Same department | -0.226 | -0.271 | 0.0962 | 0.104 | 0.0924 | 0.316 | 0.358 |
|  | (0.171) | (0.186) | (0.0495) | (0.0598) | (0.0501) | (0.116) | (0.132) |
| Same imaging technology | -0.146 | -0.171 | 0.0430 | 0.0442 | 0.0408 | 0.0461 | 0.0552 |
|  | (0.0923) | (0.0972) | (0.0321) | (0.0338) | (0.0315) | (0.0671) | (0.0712) |
| Both clinicians | -0.512 | -0.521 | 0.0255 | 0.0254 | 0.0281 | 0.0117 | 0.0154 |
|  | (0.112) | (0.115) | (0.0172) | (0.0179) | (0.0174) | (0.0591) | (0.0607) |
| Both imagers | 0.123 | 0.142 | 0.0204 | 0.0176 | 0.0208 | 0.136 | 0.124 |
|  | (0.172) | (0.177) | (0.0320) | (0.0336) | (0.0308) | (0.0929) | (0.0920) |
| Same rank | -0.457 | -0.459 | -0.0122 | -0.0112 | -0.0100 | -0.0215 | -0.0150 |
|  | (0.105) | (0.106) | (0.0148) | (0.0143) | (0.0147) | (0.0482) | (0.0467) |
| Both female | -0.367 | -0.393 | 0.00837 | 0.00867 | 0.0109 | -0.0332 | -0.0252 |
|  | (0.142) | (0.148) | (0.0223) | (0.0241) | (0.0229) | (0.0541) | (0.0684) |
| Both male | 0.229 | 0.235 | 0.0120 | 0.0126 | 0.0123 | 0.0613 | 0.0691 |
|  | (0.127) | (0.132) | (0.0143) | (0.0140) | (0.0137) | (0.0569) | (0.0565) |
| R-squared | 0.235 | 0.227 | 0.076 | 0.060 | 0.084 | 0.062 | 0.049 |

All regression models include night and room FE. Multi-way, robust standard errors in parentheses.

Table A7. OLS Regression Models of Knowledge Transfer – % of MeSH Keywords Transferred between scientist-pair {*i,j*}; N = 28,258 (Full Sample)

| VARIABLES | Model 1 | Model 2 | Model 3 | Model 4 |
| --- | --- | --- | --- | --- |
| Same room | 0.0882 | 0.0707 | 0.0537 | 0.0426 |
|  | (0.0807) | (0.147) | (0.106) | (0.159) |
| Same room x Low field sim. |  | 0.0120 |  | 0.00516 |
|  |  | (0.143) |  | (0.143) |
| Same room x Mod. field sim. |  | 0.356 |  | 0.323 |
|  |  | (0.325) |  | (0.328) |
| Same room x Low intellectual sim. |  |  | -0.0993 | -0.0975 |
|  |  |  | (0.143) | (0.144) |
| Same room x Mod. intellectual sim. |  |  | 0.199 | 0.197 |
|  |  |  | (0.135) | (0.135) |
| Low field sim. | 0.166 | 0.163 | 0.165 | 0.163 |
|  | (0.112) | (0.116) | (0.112) | (0.115) |
| Moderate field sim. | -0.0567 | -0.147 | -0.0608 | -0.142 |
|  | (0.225) | (0.212) | (0.224) | (0.212) |
| Low intellectual sim. | -4.586 | -4.586 | -4.561 | -4.561 |
|  | (0.200) | (0.200) | (0.203) | (0.203) |
| Moderate intellectual sim. | -1.026 | -1.026 | -1.075 | -1.075 |
|  | (0.146) | (0.146) | (0.153) | (0.153) |
| Night FE | Y | Y | Y | Y |
| Room FE | Y | Y | Y | Y |
| R-squared | 0.263 | 0.263 | 0.264 | 0.264 |

Multi-way, robust standard errors in parentheses.

Table A8. OLS Regression Models of Knowledge Creation – # of copublications between scientist-pair {*i,j*}; N = 28,258

(Full Sample)

| VARIABLES | Model 1 | Model 2 | Model 3 | Model 4 | Model 5 | Model 6 | Model 7 |
| --- | --- | --- | --- | --- | --- | --- | --- |
| Same room | -0.0202 | -0.0856 | -0.0508 | -0.112 | -0.0235 | -0.0240 | -0.0247 |
|  | (0.0148) | (0.0557) | (0.0300) | (0.0670) | (0.0145) | (0.0144) | (0.0143) |
| Same room x Low field sim. |  | 0.0727 |  | 0.0699 |  |  |  |
|  |  | (0.0546) |  | (0.0537) |  |  |  |
| Same room x Mod. field sim. |  | 0.166 |  | 0.162 |  |  |  |
|  |  | (0.100) |  | (0.0991) |  |  |  |
| Same room x Low intellectual sim. |  |  | 0.0388 | 0.0377 |  |  |  |
|  |  |  | (0.0267) | (0.0259) |  |  |  |
| Same room x Mod. intellectual sim. |  |  | 0.0558 | 0.0530 |  |  |  |
|  |  |  | (0.0276) | (0.0264) |  |  |  |
| Gant coapplicant |  |  |  |  | 4.030 | 4.030 | 5.206 |
|  |  |  |  |  | (1.248) | (1.248) | (1.472) |
| Coapplicant x Low field sim. |  |  |  |  | -1.388 |  | -0.471 |
|  |  |  |  |  | (1.758) |  | (1.976) |
| Coapplicant x Mod. field sim. |  |  |  |  | -3.604 |  | -4.775 |
|  |  |  |  |  | (1.302) |  | (1.531) |
| Coapplicant x Low intellectual sim. |  |  |  |  |  | -3.679 | -3.891 |
|  |  |  |  |  |  | (1.673) | (1.924) |
| Coapplicant x Mod. intellectual sim. |  |  |  |  |  | -3.789 | -3.961 |
|  |  |  |  |  |  | (1.481) | (1.803) |
| Low field sim. | -0.103 | -0.122 | -0.104 | -0.121 | -0.0844 | -0.0875 | -0.0848 |
|  | (0.0343) | (0.0460) | (0.0344) | (0.0458) | (0.0333) | (0.0342) | (0.0333) |
| Moderate field sim. | -0.0775 | -0.120 | -0.0781 | -0.119 | -0.0568 | -0.0755 | -0.0574 |
|  | (0.0387) | (0.0463) | (0.0387) | (0.0462) | (0.0390) | (0.0402) | (0.0390) |
| Low intellectual sim. | -0.0858 | -0.0858 | -0.0955 | -0.0953 | -0.0811 | -0.0733 | -0.0732 |
|  | (0.0179) | (0.0179) | (0.0227) | (0.0225) | (0.0175) | (0.0171) | (0.0171) |
| Moderate intellectual sim. | -0.0783 | -0.0785 | -0.0922 | -0.0917 | -0.0721 | -0.0653 | -0.0654 |
|  | (0.0166) | (0.0167) | (0.0211) | (0.0209) | (0.0161) | (0.0158) | (0.0157) |
| Night FE | Y | Y | Y | Y | Y | Y | Y |
| Room FE | Y | Y | Y | Y | Y | Y | Y |
| R-squared | 0.004 | 0.004 | 0.004 | 0.004 | 0.027 | 0.027 | 0.034 |

Multi-way robust standard errors in parentheses.

Table A9. OLS Regression Models of Knowledge Diffusion – # of forward citations between scientist-pair {*i,j*}; N = 28,258 (Full Sample)

| VARIABLES | Model 1 | Model 2 | Model 3 | Model 4 |
| --- | --- | --- | --- | --- |
| Same room | -0.0408 | -0.216 | -0.0201 | -0.193 |
|  | (0.0445) | (0.105) | (0.0951) | (0.134) |
| Same room x Low field sim. |  | 0.191 |  | 0.193 |
|  |  | (0.102) |  | (0.102) |
| Same room x Mod. field sim. |  | 0.577 |  | 0.575 |
|  |  | (0.328) |  | (0.326) |
| Same room x Low intellectual sim. |  |  | -0.0563 | -0.0585 |
|  |  |  | (0.0916) | (0.0915) |
| Same room x Mod. intellectual sim. |  |  | -0.00884 | -0.0171 |
|  |  |  | (0.0915) | (0.0909) |
| Low field sim. | -0.178 | -0.227 | -0.178 | -0.227 |
|  | (0.0752) | (0.0954) | (0.0753) | (0.0952) |
| Moderate field sim. | -0.134 | -0.281 | -0.135 | -0.280 |
|  | (0.114) | (0.107) | (0.114) | (0.106) |
| Low intellectual sim. | -0.288 | -0.288 | -0.274 | -0.273 |
|  | (0.0582) | (0.0582) | (0.0636) | (0.0633) |
| Moderate intellectual sim. | -0.224 | -0.224 | -0.221 | -0.220 |
|  | (0.0483) | (0.0484) | (0.0538) | (0.0535) |
| Night FE | Y | Y | Y | Y |
| Room FE | Y | Y | Y | Y |
| R-squared | 0.008 | 0.008 | 0.008 | 0.008 |

Multi-way, robust standard errors in parentheses.

Table A10. Regression Models of Knowledge Transfer - % MeSH Keywords Transferred between scientist-pair {*i*,*j*} (Low vs. High Intellectual Similarity); N = 15,817 pairs

| VARIABLES | Model 1 | Model 2 |
| --- | --- | --- |
| Same room | 0.186 |  |
|  | (0.107) |  |
| F2F communication |  | 1.480 |
|  |  | (0.842) |
| Same room x Low intellectual similarity | -0.411 |  |
|  | (0.176) |  |
| F2F comm. x Low intellectual similarity |  | -3.510 |
|  |  | (1.483) |
| Low field sim. | 0.101 | 0.149 |
|  | (0.128) | (0.129) |
| Moderate field sim. | -0.258 | -0.243 |
|  | (0.267) | (0.255) |
| Low intellectual sim. | -4.392 | -4.239 |
|  | (0.171) | (0.197) |
| Night FE | Y | Y |
| Room FE | Y | Y |
| R-squared | 0.259 | 0.245 |

Multi-way, robust standard errors in parentheses.

Note: Low intellectual similarity corresponds to 0-7 common MeSH keywords; high intellectual similarity corresponds to >7 common MeSH keywords.
